# Editorial for “Latest Advances in Diagnosis and Management of Skin Cancer”

**DOI:** 10.3390/diagnostics15101196

**Published:** 2025-05-09

**Authors:** Shi Huan Tay, Choon Chiat Oh

**Affiliations:** 1 Department of Internal Medicine, Singapore General Hospital, Singapore 169608, Singapore; tay.shi.huan@mohh.com.sg; 2 Department of Dermatology, Singapore General Hospital, Singapore 169608, Singapore

The rising global incidence of skin cancer has established this disease as a critical public health issue. In recent years, numerous technological innovations have enabled more precise and timely diagnoses, while novel treatments have emerged for advanced stages of the disease. To highlight these significant developments, this Special Issue presents 11 valuable contributions to the field—ranging from detailed observations of rare and atypical cases to crucial advancements in diagnostics, treatment, and artificial intelligence (AI)-assisted decision-making.

The future of skin cancer diagnoses is increasingly poised to incorporate AI-assisted technologies, as the field moves toward integrating multi-modality data from global sources [1]. One study in our Special Issue explored Explainable AI (XAI) using ABELE for skin lesion classification. The saliency maps generated by ABELE provided useful topographical information that enhanced physicians’ confidence in their diagnoses. To realise ABELE’s potential, the training dataset must be expanded to include a broader range of lesion types, disease stages, and patient demographics. Future international collaborations will be essential for advancing our knowledge amidst increasingly complex experimental designs and analytical methodologies.

Beyond the promise of AI, the field of cutaneous melanoma has also witnessed advances in bedside, histopathological, and molecular diagnostics [2,3]. In particular, novel imaging techniques have emerged to improve early melanoma diagnosis. D’Onghia et al. highlighted the strengths of high-magnification dermoscopy and fluorescence-assisted videodermatoscopy in a study involving 85 patients with facial skin lesions [4]. Alongside the advent of line-field confocal optical coherence tomography [5], we are one step closer to untangling this Gordian knot non-invasively, potentially reducing the need for biopsies.

The emergence of programmed cell death-1 (PD1) inhibitors has transformed the management of metastatic melanoma; however, clinicians must remain vigilant regarding immune-related adverse events [6]. Another study in our Special Issue contributes a Southeast Asian perspective to the growing body of evidence on anti-PD1 inhibitors. Consistent with findings from other parts of the world, these inhibitors were shown to increase overall median survival, although a subset of melanoma patients developed cutaneous adverse reactions. Interestingly, the development of such reactions may be associated with improved overall survival, underscoring the need for future mechanistic work to unravel the underlying intricacies.

Cellular therapy represents the next frontier in skin cancer treatment, offering tailored therapeutic potential for patients who are unresponsive to current standard-of-care treatment. Indeed, several landmark trials involving tumour-infiltrating lymphocytes (TILs) and personalised, neoantigen-specific autologous T cells have demonstrated significant efficacy and safety in advanced melanoma refractory to immune checkpoint inhibitors (ICIs) and BRAF/MEK inhibitors [7,8]. Additionally, T cell-based vaccines against commonly occurring β-human papillomaviruses (β-HPVs), along with β-HPV-specific T cell immunotherapy, are also promising strategies in the fight against HPV-related cutaneous squamous cell carcinoma (cSCC).

All in all, we are witnessing a paradigm shift in skin cancer, marked by multiple breakthroughs across diagnostics and therapeutics. We hope this Special Issue will inspire continued contributions to the scientific understanding and clinical management of skin cancer, ultimately advancing patient care worldwide.
